# Anatomy, Descriptive and Surgical

**Published:** 1862-04

**Authors:** 


### Book Notices.

ANATOMY, Descriptive and Surgical. By HENRY GRAY, F. R. S., and Lecturer on Anatomy at St. George's Hospital Medical School. The Drawings by H. V. CARTER, M. D., late Demonstrator of Anatomy at St. George's Hospital. The Dissections jointly by the Author and Dr. CARTER. Second American, from the revised and enlarged London Edition. With Three Hundred and Ninety-Five Engravings on Wood. Imperial octavo. Pp. 816. Philadelphia: BLANCHARD & LEA. 1862.

Time robs many of our adages and proverbs of much of their significance; but no one of these morsels of *word-pemmican* loses so much, year by year, as the one which denies the existence of a "royal road to knowledge." Here, for example, is a volume, by the casual turning over of whose pages, for a desultory hour, the least diligent of students will absolutely *absorb* more anatomical knowledge than was formerly vouchsafed to weary hours of patient study of the dull and dignified text-books of our youth.

To those who, as teachers or students, are already familiar with this work, words of commendation would be superfluous. Upon the appearance of the first edition, we took great pleasure in commending it to the profession, as a book of superior claims,—in fact, the best of the text-books on Anatomy. We were confident that a work so eminently adapted to the wants of our American students would be at once appreciated—and the appearance of a second edition in so brief a space of time, is the most conclusive evidence that we were not mistaken.

For the benefit of those who have not yet had the good fortune to meet with "GRAY's," we may briefly sum up its most valuable points, as follows:—first, and most palpable,—the *illustrations*; these, aside from the fact that they are entirely original, and drawn by Dr. CARTER directly from dissections made by him and Mr. GRAY, embody a distinctive feature, in printing upon each part the name of the object itself, instead of the old plan of numbers and foot-notes,—a plan which not seldom makes confusion worse confounded in the tyro's mind. Osteology, by this mode of accurately-lettered engravings, becomes a pastime, and the temporal bone, with its puzzling petrous portion, becomes a permanent fixture in the mind's eye of the student. Not to offend Mrs. Malaprop, we abstain from comparisons; but the reader may make them for himself as between GRAY and any of the older standard works,—taking, for example, the illustrations of the articulations of the atlas with the axis, and with the occipital bone. *

Secondly, we notice the union of descriptive and regional anatomy,—the small-text remarks on the surgical importance and relations of each part as it comes up for description,—thus presenting to the student the *end* of his labors, while still engaged in mastering the *means*,—holding out to him an inducement for thorough work, showing him the practical value of his studies.

Thirdly, the amount of valuable surgical information, presented in such an apposite manner and conjunction as to relieve it of any appearance of trenching on the province of the surgical text-book, and yet conveying to the student hints and knowledge he might search for in vain in the latter.

Fourthly, its value in the dissecting-room, full and complete, even in the most minute particulars.

In this new edition, we notice one or two corrections, and such a careful revision of the text, and addition, both to the matter and illustrations, as to bring the volume fully up with the recent improvements in surgery.

---

* Page 192, *et seq.* 2d Edition.

